# Plant-Based Family Food Packages and Weight Change in Children During the COVID-19 Pandemic

**DOI:** 10.5888/pcd20.220336

**Published:** 2023-06-22

**Authors:** Allison J. Wu, Jacob B. Mirsky, Meghan E. Perkins, Mandy Luo, Cara F. Ruggiero, Michael Lenson, Rachel Burgun, Elsie M. Taveras, Lauren Fiechtner

**Affiliations:** 1 Division of Gastroenterology, Hepatology, and Nutrition, Boston Children’s Hospital, Boston, Massachusetts; 2 Harvard Medical School, Boston, Massachusetts; 3 Division of General Internal Medicine, Massachusetts General Hospital, Boston, Massachusetts; 4 Division of General Academic Pediatrics, Massachusetts General Hospital for Children, Boston, Massachusetts; 5 The Greater Boston Food Bank, Boston, Massachusetts; 6 Department of Nutrition, Harvard T. H. Chan School of Public Health, Boston, Massachusetts; 7 Division of Gastroenterology and Nutrition, Massachusetts General Hospital for Children, Boston, Massachusetts

## Abstract

Food insecurity and obesity coexist among children and families. We examined the association between receipt of plant-based family food packages from the Massachusetts General Hospital Revere Food Pantry and change in body mass index (BMI), adjusted for age and sex, among children during the COVID-19 pandemic. Among 35 children aged 2 to 18 years who received the packages between January 2021 and February 2022, we observed a change in BMI of −0.04 kg/m^2^ (95% CI, −0.08 kg/m^2 ^to −0.01 kg/m^2^) for each package received. Our results suggest plant-based food packages might mitigate, and potentially reverse, BMI increase in children in households seeking food assistance.

**Summary**

**What is already known on this topic?**

The prevalence of childhood obesity has increased in the US during the COVID-19 pandemic along with social needs, including food insecurity.

**What is added by this report?**

We examined changes in body mass index (BMI) among children in households that received weekly plant-based family food packages during the pandemic from the Massachusetts General Hospital Revere Food Pantry. We observed that increasing receipt of food packages was associated with a decrease in BMI among 35 children aged 2 to 18 years.

**What are the implications for public health practice?**

Our results suggest that providing healthy plant-based food packages might mitigate or reverse increases in BMI among children requiring food assistance.

## Objective

The prevalence of childhood obesity in the US increased from 19.3% to 22.4% during the first stages of the COVID-19 pandemic (August 2019–August 2020) (1). In Massachusetts, obesity prevalence among children and adolescents increased from 15.1% in 2018 to 15.7% in 2019 and 17.3% in 2020 (2). Food insecurity increased by 55% in 2020 to roughly 1.6 million adults and affected 42% of households with children (3). These public health threats have disproportionately affected Black, Latino, and low-income families.

In adults, food insecurity is associated with poor dietary quality (4), which is further associated with development of obesity, heart disease, diabetes, and certain cancers (5). While evidence of associations between food insecurity and dietary quality in children is mixed, increasing attention is being given to nutrition security, defined as “having equitable and stable availability, access, affordability, and utilization of foods and beverages that promote well-being and prevent and treat disease” (6). Improving nutrition security in childhood is critical to preventing chronic disease. High consumption of plant-based foods has been shown to prevent and treat cardiovascular (7) and other chronic diseases. We therefore aimed to examine the association of receipt of plant-based family food packages with weight change in children.

## Methods

**Figure 1:**
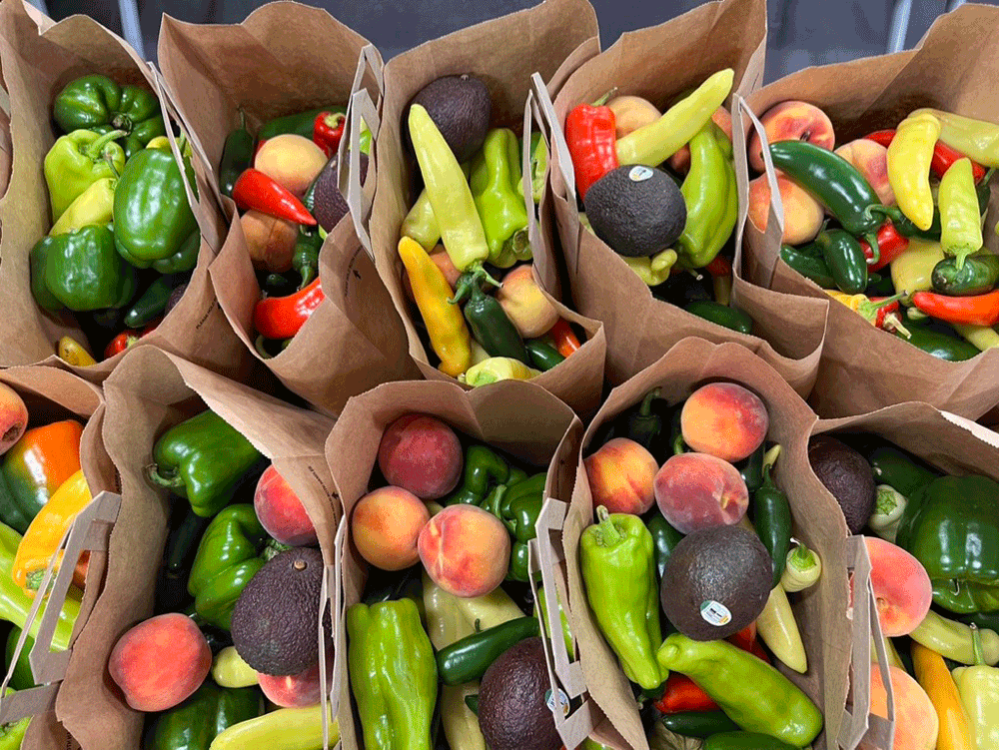
Photograph of foods included in the Massachusetts General Hospital Revere Food Pantry plant-based family food packages. Image from Jacob B. Mirsky, MD, and reprinted with permission.

The Massachusetts General Hospital (MGH) Revere Food Pantry is based in an academic hospital clinic. Details of the food pantry’s plant-based food approach and partnership between MGH and the Greater Boston Food Bank have been published (8). The Greater Boston Food Bank and a local gleaning organization supplied the food pantry. All patients from MGH Revere Food Pantry seeking food assistance were eligible to receive weekly plant-based family food packages. Packages included fresh fruits and vegetables (Figure 1), nuts, and whole grains and were adjusted for family size to provide 3 meals per day for each household member. The food pantry’s registered dietitian ensured the protein provided by the packages was aligned with dietary guidelines.

**Figure 2:**
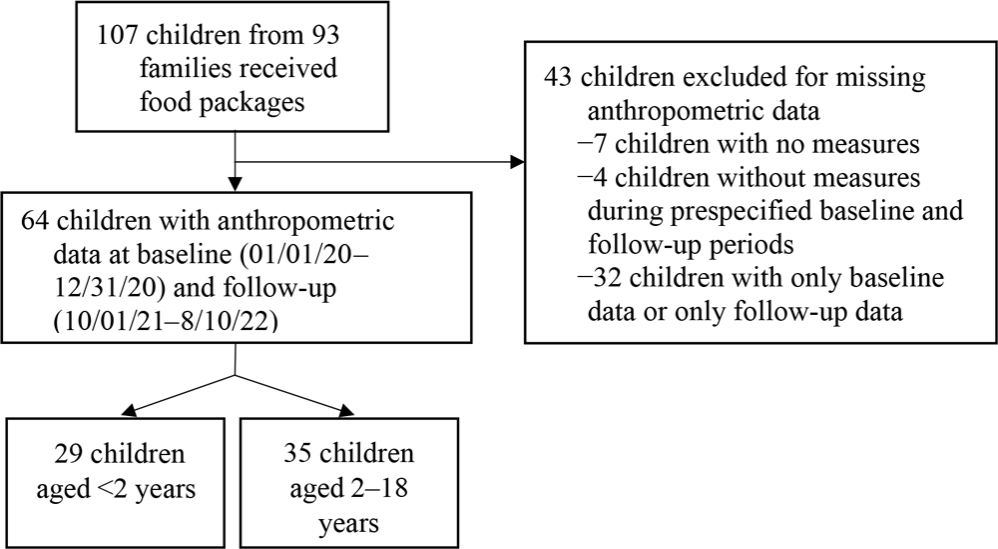
Flow chart describing selection of children aged 18 years or younger from 93 households participating in the Massachusetts General Hospital Revere Food Pantry program.

Among 93 families who received family food packages, 107 children (aged ≤18 y at baseline) had electronic health record data available (Figure 2). We restricted our analyses to children (n = 64) with length or height and weight measurements at both baseline (January 1–December 31, 2020) and follow-up (October 1, 2021–August 10, 2022). For children younger than 2 years (n = 29), we calculated the *z* score for change in weight-for-length. For children aged 2 to 18 years (n = 35), we examined change in absolute BMI and age- and sex-adjusted BMIp_95 _(percentage of the 95th percentile BMI) from baseline to follow-up. We used linear regression to examine the association between total number of family food packages received and change in the weight-for-length *z* score for children younger than 2 years and BMI and BMIp_95_ for children aged 2 to 18 years between baseline and follow-up. For absolute BMI, we adjusted for child age, sex, and duration of time between BMI measures. For weight-for-length *z* score and BMIp_95_, we adjusted for duration of time between BMI measures. The Institutional Review Board of MGH approved the study. We conducted all analyses using R Studio, version 4.1.0 (Posit Software).

## Results

Between January 1, 2021, and February 1, 2022, 107 children from 93 families received weekly family food packages. On average, households received 27.4 (SD, 18.1) packages during that period. The mean household size was 4.6 people (SD, 1.4) with an average of 2.2 (SD, 0.9) children per household. Among children with 2 weight-for-length *z* scores or BMI measures (n = 64), 23 (36%) children identified as Hispanic, 8 (13%) children as non-Hispanic White, 3 (5%) children as non-Hispanic Black, 10 (16%) children as other (including Asian); 20 (31%) children were missing data on race or ethnicity (Table 1). Sixty-one children (95%) had public health insurance (eg, MassHealth).

**Table 1:** Characteristics of Infants and Children With Electronic Health Record Data (N = 64) in Households Who Received Family Food Packages From the Massachusetts General Hospital Revere Food Pantry, January 2021–February 2022^a^

Characteristic	Infants (<2 y) (n = 29)	Children (2–18 y) (n = 35)
Race or ethnicity, n (%)^b^		
Non-Hispanic White	2 (6.9)	6 (17.1)
Non-Hispanic Black	0	3 (8.6)
Hispanic	12 (41.4)	11 (31.4)
Other	1 (3.4)	9 (25.7)
Data missing	14 (48.3)	6 (17.1)
**Insurance, n (%)**		
Public	26 (89.7)	35 (100)
Private	3 (10.3)	0
**Baseline visit, 1/1/2020–12/31/2020**		
Age, y	1.1 (0.5)	7.2 (4.3)
Weight-for-length *z* score	−0.05 (1.00)	NA
Weight-for-length ≥97.7 percentile, n (%)	1 (3.4)	NA
BMI	NA	19.9 (4.6)
BMI percentile	NA	79.8 (23.3)
BMIp_95_, %	NA	96.2 (16.0)
Overweight or obesity, n (%)^c^	NA	20 (57.1)
**Follow-up visit, 10/1/2021–8/10/2022**		
Age, y	2.6 (0.6)	8.7 (4.5)
Weight-for-length* z* score^b^	0.66 (0.99)	NA
Weight-for-length ≥97.7 percentile, n (%)	10 (34.5)	NA
BMI	NA	20.7 (5.5)
BMI percentile, %	NA	75.6 (25.9)
BMIp_95_	NA	95.9 (18.1)
Overweight or obesity, n (%)^c^	NA	17 (48.6)
**Change from baseline to follow-up**		
Time between visits, months	18.6 (3.2)	18.5 (6.5)
Weight-for-length *z* score	0.72 (0.93)	NA
BMI adjusted for age and sex	NA	0.31 (0.41)
BMIp_95_	NA	−0.3 (7.9)

Abbreviations: BMI, body mass index; BMIp_95_, percentage of the 95^th^ percentile body mass index adjusted for age and sex; NA, not applicable.

a Values are mean (SD) except where otherwise indicated.

b Among children aged 0–18 years overall with 2 weight-for-length *z* scores or BMI measures (n = 64), 23 (36%) children identified as Hispanic, 8 (13%) children as non-Hispanic White, 3 (5%) children as non-Hispanic Black, 10 (16%) children as other (including Asian); 20 (31%) children were missing data on race or ethnicity.

c Overweight/obesity was defined as BMI ≥85^th^ percentile for children.

Overall, children younger than 2 years experienced a mean change of 0.72 (SD, 0.93) units in weight-for-length *z* score, while children aged 2 to 18 years experienced a 0.31 (SD, 0.41) kg/m^2^ change in age- and sex-adjusted BMI (Table 1). The mean duration between baseline and follow-up measures was 19 months. At baseline, 20 (57%) children aged 2 to 18 years were classified as having overweight or obesity, defined as a BMI at or above the 85^th^ percentile, per CDC standardized growth charts (9). At follow-up, 17 (49%) children were classified as having overweight or obesity. Among children younger than 2 years, we found no significant association (*P *= .61) between total family food packages received and change in weight-for-length *z* score. For children aged 2 to 18 years, per each additional package received, we saw an associated change in BMI of −0.04 kg/m^2^ (95% CI, −0.08 kg/m^2 ^to −0.01 kg/m^2^) from baseline to follow-up, independent of age, sex, and duration between measures (Table 2). Per each additional family food package received, we saw no significant association (*P *= .12) in change in BMIp_95_.

**Table 2:** Association Between Number of Food Packages Received and Change in Weight-for-Length, BMI and BMIp_95_ Among Infants and Children With Electronic Health Record Data (N = 64) in Households Who Received Family Food Packages From the Massachusetts General Hospital Revere Food Pantry, January 2021–February 2022

Variable	Per additional food package received	
	β (95% CI)	*P* value
**Infants <2 y (n = 29)**		
Change in weight-for-length *z* score^a^	−0.01 (−0.03 to 0.02)	0.61
**Children 2–18 y (n = 35)**		
Change in BMI^a^	−0.04 (−0.08 to –0.01)	0.03
Change in BMIp_95_ a	−0.13 (−0.29 to 0.03)	0.12

Abbreviations: BMI, body mass index; BMIp_95_, percentage of the 95^th^ percentile body mass index adjusted for age and sex.

a Adjusted for time between measurements. BMI models are additionally adjusted for age and sex.

## Discussion

In our study, weight-for-length *z* score and BMI increased from baseline (2020) to follow-up during the COVID-19 pandemic (October 2021–August 2022). These increases were consistent with nationwide data (1,10). We did not observe an association between total number of family food packages and change in the weight-for-length score among children younger than 2 years. With each additional family food package received, we observed an associated decrease in BMI among children aged 2 to 18 years. We estimate that a child in the households we studied who received 27 weeks or more of packages might have a BMI decrease of 1.08 kg/m^2^ or more. Because children in the US experienced increases in BMI during the pandemic, a decrease in BMI observed in our study among children receiving an increased number of family food packages is notable. Per the US Preventive Services Task Force, arresting weight gain is a clinically important outcome for many interventions (11).

To our knowledge, our study is among the first to examine the association between a nutrition security intervention and weight changes in children during the COVID-19 pandemic and the burgeoning “food is medicine” movement (ie, efforts to integrate food-based nutrition interventions into health care systems). A 6-month prescription produce program that increased fruit and vegetable intake among young Navajo children from 2015 through 2018 showed a decrease in BMI percentile among children (n = 58) who were overweight or had obesity at baseline, from an average of the 95.6 to 73.1 percentile (12).

Limitations to this case study include its small sample size, coming from MGH Revere Food Pantry, a single health center. We examined weight change within, and not between, people. Our study was likely underpowered to detect associations among infants. Because of our inclusion criteria, which relied on linkage to the health record, participants were required to be MGH patients. There may be unmeasured confounding (eg, severity of food insecurity, presence of other unmet social needs). We did not have the food pantry referral date to identify exact pre- and postintervention periods. Our case study is unable to specify how plant-based packages, and which familial factors, helped children achieve improvement in BMI. 

The results of our case study suggest that providing plant-based family food packages could be a useful strategy to prevent, and potentially reverse, BMI increases among children requiring food assistance. Food pantry packages should be considered in conjunction with other healthy-weight interventions for children.
